# A microfluidic bone marrow model combining CFD and organ-on-a-chip technologies to study leukemia niche dynamics

**DOI:** 10.3389/fbioe.2026.1766296

**Published:** 2026-05-28

**Authors:** Gabriel Santos Rosalem, Diego Rodney Rodrigues De Assis, Libardo Andrés González Torres, Estevam Barbosa de Las Casas, Wagner Nunes Rodrigues, Rafael Silva Gonçalves, Rayane Aparecida Nonato Rabelo, Jeronimo Conceição Ruiz, Maria Gabriela Reis Carvalho

**Affiliations:** 1 Rene Rachou Institute, Oswaldo Cruz Foudation, Belo Horizonte, Brazil; 2 Institute of Science and Technology, Universidade Federal dos Vales do Jequitinhonha e Mucuri, Diamantina, Brazil; 3 Structural Engineering Department, Universidade Federal de Minas Gerais, Belo Horizonte, Brazil; 4 Department of Physics, Universidade Federal de Minas Gerais, Belo Horizonte, Brazil

**Keywords:** 3D printing, microphysiological system, bone marrow, computational simulation, leukemia, niches, organ-on-a-chip

## Abstract

**Introduction:**

B-cell acute lymphoblastic leukemia (B-ALL) disrupts the architecture and function of the bone marrow niche. However, current in vitro and in vivo models fail to fully capture the spatial, biochemical, and mechanical complexity of the native microenvironment. Here, we present a biomimetic bone marrow on-a-chip that integrates organ-on-a-chip technology, 3D hydrogel culture, and computational modeling to recreate the perivascular, central, and endosteal niches of human bone marrow.

**Methods:**

The Computational Fluid Dynamics (CFD) was used to guide the design and operation of the microdevice by predicting physiological interstitial flow within the culture system, enabling consistent mechanical stimuli as in vivo under conditions compatible with bone marrow physiology. The microdevice was fabricated using high-resolution 3D printing and soft lithography, and incorporates phaseguide structures for hydrogel confinement, the establishment of three distinct niches and continuous perfusion. Co-cultures of endothelial, stromal, osteoblast, and leukemic cells were maintained in a type I collagen matrix under dynamic conditions.

**Results/Discussion:**

The platform supported high cell viability and enabled compartmentalized spatial organization of multicellular co-cultures. The presence of leukemic cells was associated with changes in soluble signaling molecules within the microenvironment, including increased levels of cytokines, chemokines, and growth factors such as IL-10, IL-13, TNF-α, CCL2, CCL3, CCL5, FGF, G-CSF, and GM-CSF. These patterns are consistent with signaling processes linked to immunoregulation, leukemic supportive signaling, and therapeutic resistance in B-ALL.

**Conclusion:**

Together, these findings indicate that the bone-marrow-on-a-chip captures relevant aspects of niche-associated signaling and provides a versatile platform for investigating leukemia microenvironment interactions, with potential in drug screening and preclinical model development.

## 1 Introduction

Human hematological disorders resulting from a combination of genetic events and environmental factors can lead to pathologies that disrupt the hematopoietic processes of the bone marrow (Franco and Godley, 2025). One of the main pathologies is leukemia, characterized by a block in the differentiation process of blood cells at a proliferative stage. It generates abnormal, or immature, cells called blasts or leukemic stem cells, which continuously expand throughout the bone marrow (Dander et al., 2021). Among the most frequent variants of leukemia is acute lymphoblastic (ALL), marked by aberrant proliferation and differentiation of B (B-ALL) and T (T-ALL) lymphocytes in bone marrow niches. B-ALL disorders account for 80% of all cases (Chiarini et al., 2016). This pathology represents the most common childhood leukemia, affecting children between 2 and 9 years of age, while adult cases are generally associated with a more aggressive clinical course and poorer prognosis (Penchansky, 2003; Terwilliger and Abdul-Hay, 2017; Garcia-Gimenez and Richardson, 2023). As B-ALL cells expand, they progressively occupy bone marrow niches, hijacking the microenvironment and thereby inhibiting the production of healthy blood cells, ultimately disrupting normal hematopoiesis (Wang and Hasserjian, 2018; Hughes et al., 2022).

Within the bone marrow, the complexity and heterogeneity of the microenvironment are often conceptualized as a spatially partitioned niche, namely, the perivascular, central, and endosteal, each characterized by distinct cellular composition, a structured extracellular matrix (ECM), and soluble mediators (Raic et al., 2019; Belyavsky, 2019; Kandarakov et al., 2022). These niches orchestrate the structural and biochemical microenvironment that underlies both healthy hematopoiesis and leukemic progression (Dander et al., 2021; Xiao et al., 2022), playing a critical role in regulating cell survival, proliferation, and chemoresistance of leukemic cells, thus affecting the response to treatment (Panting et al., 2024).

The spatial organization and biochemical complexity of the human bone marrow make it inherently difficult to investigate its pathophysiology and to accurately evaluate therapeutic pharmacological responses, thereby motivating the development of alternative *in vitro* platforms (Huh et al., 2011).

In traditional culture methods, a crucial limitation is the inadequate biomimicry of a physiologically relevant microenvironment, which results in low-efficiency therapeutic approaches (Esch et al., 2015). Bidimensional (2D) and three-dimensional (3D) platforms often fail to reproduce both the microarchitectural complexity of the cellular environment and the physicochemical gradients generated by flow over time and space (Duval et al., 2017). On the other hand, animal models frequently fail to accurately replicate human pathophysiology, hindering the extrapolation of experimental results and predictions (Shanks et al., 2009). Furthermore, from an ethical standpoint, there is a global movement to reduce animal testing through the implementation of alternative methods (Olsson et al., 2017).

Defined as “microfabricated, integrative platforms designed to recapitulate *in vitro* the functional units of human organs” (Bai and Wang, 2020), Organ-on-a-Chip (OoC) microdevices have emerged as leading alternatives in cell culture methodologies. Given the central role of the bone marrow in health and hematological malignancies, there is a growing scientific interest in mimicking its functions and niches through the use of an OoC platform.

Recent advances in bone marrow-on-a-chip models to study ALL cancer have led to the development of more reliable biomimetic microenvironments *in vitro*, enhancing the understanding of the pathophysiological functions and fate of bone marrow cells (Chou et al., 2020; Ma et al., 2020; Nelson et al., 2021; Glaser et al., 2022). These works contribute to the development of methodologies that take into account the genetic profile of each patient or the use of medications that interact in a more specific pathway, thereby minimizing adverse effects in humans. However, most models do not biomimetically reproduce all essential characteristics, which include physically integrated continuous culture chambers, co-culture of the main cell types on health/leukemic environment, simulation of stimuli induced by interstitial flow, establishment of biochemical gradients across niches, and consideration of distinct mechanical properties for each of these niches (Rosalem et al., 2020). Therefore, gaps still exist *in vitro* representation of microenvironment tumors in bone marrow.

Aiming to advance the understanding of bone marrow pathophysiology, we developed a microfluidic bone marrow-on-a-chip platform that integrates CFD simulations with experimental validation into a physiologically biomimetic system. The platform was designed to recapitulate key structural and transport features of the native marrow microenvironment, including interstitial flow regimes and spatially constrained cellular organization, and their impact on leukemia niche dynamics. We demonstrate that this platform reproduces key aspects of bone marrow niche organization, including spatial confinement, fluid-mediated stimulus, and stromal-leukemic interactions.

## 2 Materials and methods

### 2.1 Organ-on-a-chip microfabrication

This study employed an OoC designed to recapitulate physically integrated marrow niches under physiologic interstitial fluid flow. The microdevice comprises microchambers (1000 μm wide and 500 μm high) representing distinct niches, interconnected by phaseguide structures that enable independent hydrogel loading while preventing inter-compartmental leakage. This configuration allows niche-specific cell culture within an integrated perfused system.

The OoC platform was fabricated using a combination of additive manufacturing and soft lithography replica molding technique. A master mold was designed with SolidWorks 2024 (Dassault Systèmes SolidWorks Corp.), exported as Standard Tessellation Language (STL) file, and printed with a high-precision DLP 3D printer (Profluidics 285D, CADWorks3D) using a microfluidic-specific resin (CW3D-R-MMG-500G; Master Molder Resin, CADWorks3D). The STL file was processed (Utility Ver 6.4.4. t12, CADWorks3D) following optimized parameters: layer thickness, 10 μm ; gap adjustment, 0 mm; base layer, 1; base curing, 40 s; buffer layer, 2; power, 100%; print delay, 65 s.

Printed molds were washed twice in isopropyl alcohol (Merck, 1096341000) for 15 min in an ultrasonic bath at 100 W, followed by manual drying with an airbrush loaded with isopropanol for approximately 1 min. Post curing was performed in a Cure Zone chamber (CW3D-A-PCZ; CADWorks3D) at 40.0 $mW/{cm}^{2}$ for 40 min. A homogeneous mixture of polydimethylsiloxane (PDMS) elastomer and curing agent (Sylgard 184 kit, Dow Corning) at a 10:1 weight-to-weight (w/w) ratio was degassed under vacuum at 7.25 psi for 30 min to remove air bubbles, and then poured over the master mold. The set-up was cured at 80 °C for 2 h, peeled from the mold, and port holes (1.5 mm for hydrogel and 3 mm for fluidics) were created with biopsy punches.

The PDMS layer was cleaned in an ultrasonic bath with isopropanol and distilled water (5 min each), then bound to a 24 mm × 50 mm glass coverslip, via oxygen plasma. PDMS layer surface with microfluidic pattern and the glass coverslip were exposed to oxygen plasma at 150 mTorr, 18 W for 50 s in a Harrick Plasma oven (PDC-32G-2; Basic Plasma), immediately brought into contact and then incubated at 80 °C to complete the assembly.

Sterilization was performed under a class II laminar flow hood, by filling the microchannels with 70% ethanol, rinsing three times with sterile distilled water, and drying overnight at room temperature. Finally, the internal surfaces of the sterilized OoC were functionalized with dopamine hydrochloride (Sigma-Aldrich, H8502) at 2 mg/mL for 2 h at room temperature to promote ECM adhesion. The chip was then washed with sterile deionized water, and dried in an oven at 60 °C overnight.

### 2.2 Computational microfluidics

A computational model was developed to simulate and predict interstitial fluid flow within a porous hydrogel medium in the microdevice. The control volume geometry was designed in SolidWorks 2024 (Dassault Systèmes SolidWorks Corp.) and implemented in the finite element software COMSOL Multiphysics (v5.6). The governing discretized equations were solved under steady-state conditions on a high-performance Linux workstation.

Mesh convergence analysis was performed using the Grid Convergence Index method (GCI) (Equations 1–3) (Carvalho et al., 2024), combined with skewness metrics (Equation 4) (COMSOL Inc, 2019):

$${GCI}_{\text{mesh}}^{ji}=\frac{1.25e_{a}^{ji}}{r_{ji}^{\text{p}}-1}$$ (1)

$$e_{a}^{ji}=\left|\frac{\phi_{j}-\phi_{i}}{\phi_{j}}\right|$$ (2)

$$p=\frac{1}{ln\left(r_{ji}\right)}\left|ln\left|\frac{\epsilon_{ji}}{\epsilon_{kj}}\right|\right|$$ (3)

$$Skewness=\left\{\begin{array}{cc} 1\text{,} & \text{perfectly regular element}\\ \text{0,} & \text{degenerated element} \end{array}\right.$$ (4)

where *i*, *j*, and *k* represent the index for predefined mesh (3 – *Extra Fine*; 2 – *Finer*; 1 – *Fine*), $r_{ji}$ the grid refinement factor, $e_{a}^{ji}$ relative error, p apparent order, $\phi_{i}$ the computed velocity at each mesh, and $\epsilon_{ji}$ the difference between velocity $\phi_{j}$ and $\phi_{i}$ . The finite element mesh selected for simulations was *Finer* physics-controlled mesh, composed by 1,531,133 free tetrahedral domain elements. A 3D stationary regime was used to study the fluid velocities. The fluid was considered incompressible and Newtonian, with flow behavior in a free channel (Poon, 2022). It was described by the Stokes equations: continuity (Equation 5) and conservation of linear momentum (Equation 6), neglecting inertial terms (creeping flow). To model the flow within porous hydrogel compartments, Brinkman’s equation was employed (Equation 7):

ρ∇⋅u=0 (5)

$$\rho \left[\frac{\partial u}{\partial t}\right]=\nabla \cdot \left[-PI+\mu \left(\nabla u+{\left(\nabla u\right)}^{\text{T}}\right)\right]$$ (6)

$$\frac{\rho }{\varepsilon_{\text{p}}}\left[\frac{\partial u}{\partial t}\right]=\nabla \cdot \left[-PI+\frac{\mu }{\varepsilon_{\text{p}}}\left(\nabla u+{\left(\nabla u\right)}^{\text{T}}\right)-\frac{2}{3}\mu \frac{1}{\varepsilon_{\text{p}}}\left(\nabla \cdot u\right)I\right]-\left(\frac{\mu }{\kappa }\right)u$$ (7)

where $u\;\left[m/s\right]$ denotes the fluid velocity vector, $\rho \;\left[kg/m^{3}\right]$ the specific mass of the fluid, $P\;\left[Pa\right]$ the pressure, $\mu \;\left[N.s/m^{2}\right]$ the fluid dynamic viscosity, $\varepsilon_{\text{p}}$ the porosity, $\kappa \;\left[m^{2}\right]$ the permeability of the porous medium, and **I** the identity tensor.

The boundary conditions for the computational simulation were defined as follows: (1) at the left fluid port, a syringe pump-driven interstitial fluid flow was defined as inlet with a constant flow rate of 0.05 μL/min ; (2) at the right fluid and hydrogel filling ports, closed boundaries were imposed (no flow); (3) atmospheric pressure was assumed at the outlet; and (5) a no-slip condition $\left(u=0\right)$ was assumed on all inner microchannels walls.

The physical properties of the fluid were defined based on standard values for culture medium at 37 °C , with an assumed density of 1007 $kg/m^{3}$ and dynamic viscosity of $9.58\times 10^{-4}\;N.s/m^{2}$ (Poon, 2022). The porous medium compartment was modeled as a 3D type I collagen matrix at a concentration of 2.4 mg/mL , with a permeability of $10^{-12}\;m^{2}$ and a porosity of 0.8015 (Moreno-Arotzena et al., 2015).

The interstitial fluid velocity distribution was evaluated across virtual lines spanning all culture chambers and phaseguide structures. The resulting flow patterns were visualized using heat maps and streamline plots of velocity.

### 2.3 Cell culture

Human cells were maintained under standard culture conditions (37 ° C, 5% ${\text{CO}}_{2}$ , and 95% humidity). The stromal cells (ATCC, CRL- $11882^{\text{TM}}$ ; Hs-5 cell line) were cultured in RPMI 1640 medium (Gibco, 23400021), supplemented with 10% fetal bovine serum (FBS) (Gibco, 12657029), 1% penicillin/streptomycin (Sigma, 102432), and 1% ${\text{GlutaMAX}}^{\text{TM}}$ (Gibco, 35050061). Endothelial cells (BCRJ, 0345; EA. hy926) were cultured in high-glucose DMEM medium (Gibco, 11965092), supplemented with 10% FBS, 1% penicillin/streptomycin, and 1% ${\text{GlutaMAX}}^{\text{TM}}$ . Osteoblasts (BCRJ, 0217; Saos-2 cell line) were cultured in DMEM/F12 medium (Gibco, 11039021), supplemented with 15% FBS, 1% penicillin/streptomycin, 1% ${\text{GlutaMAX}}^{\text{TM}}$ , and 1 mM sodium pyruvate (Gibco, 11360-070). The B-ALL REH cell line, kindly provided by Dr. Dario Campana (St. Jude Children’s Research Hospital, United States), was cultured in RPMI 1640 medium, supplemented with 20% FBS, 1% penicillin/streptomycin, 1% ${\text{GlutaMAX}}^{\text{TM}}$ , 1 mM sodium pyruvate, and 2.5 g/L dextrose (Êxodo Científica, D06816RA).

### 2.4 Cell loading, hydrogel encapsulation, and 3D culture

Niche-specific cells were introduced into the OoC using a stepwise loading protocol. Osteoblasts and endothelial cells, both at $2\times 10^{6}\;cells/mL$ , were seeded simultaneously into their respective chambers. Osteoblasts were suspended in type I rat tail collagen (Gibco, A10483-01), and 15 μL of the mixture were loaded into the endosteal chamber via the hydrogel port. In parallel, endothelial cells were mixed with 0.2 mg/mL Geltrex (Gibco, A1413202), and 15 μL were seeded into the perivascular chamber. To improve seeding homogeneity, cells were suspended in a collagen-containing pre-gel solution during loading. While compartmentalization is governed by phaseguide structures, this approach supports uniform cell distribution and enables initial cell–surface interactions without pre-coating. The increased viscosity also stabilizes flow during loading, reducing cell loss due to flushing.

Ports were sealed with adhesive tape ( ${3\text{M}}^{\text{TM}}$ , 9795R) to prevent evaporation and leakage. The microdevices were then inverted and incubated overnight under standard culture conditions (37 ° C, 5% ${\text{CO}}_{2}$ , and 95% humidity) to promote cell adhesion. Subsequently, culture medium and non-adherent cells were removed.

To recapitulate the 3D marrow matrix, leukemic and stromal cells were embedded within 2.5 mg/mL type I bovine skin collagen hydrogel (Sigma-Aldrich, 5074). Two experimental configurations were established: a) Control condition: stromal (Hs-5) at $5\times 10^{5}\;cells/mL$ ; b) Pathological condition: co-encapsulation of REH and stromal cells at 3:1 ratio (REH:Hs-5), with a total final cell density of $2\times 10^{6}\;cells/mL$ in the final solution. This ratio was selected to reflect the high leukemic burden at diagnosis. Clinical studies have reported that blasts ranged from 20% to 80% in bone marrow (Pandey, 2021). A volume of 15 μL of collagen/cells mixture was injected into the endosteal chamber through the hydrogel port, and solidified at 37 ° C, 5% ${\text{CO}}_{2}$ , and 95% humidity for 30 min. Then, 15 μL of solution was injected into central and perivascular chambers and incubated for 50 min.

Fluidic channels were filled with 100 μL of 1:1:1:1 culture medium mix (RPMI 1640 10% FBS, RPMI 1640 20% FBS, high glucose DMEM, and DMEM/F12). After 24 h of static culture for stabilization, hydrogel ports were sealed with adhesive tape and interstitial fluid flow was generated via a syringe pump (Pump 33 DDS, Harvard Apparatus) at 72 μL/day . The hydraulic system integrated a 1 mL syringe filled with the mix of culture medium connected to the fluidic port inlet and a 500 μL conical bottom microtube to the fluidic port outlet, both via silicone tubing. The conical microtube was used to collect the effluent during culture. The dynamic co-culture was maintained for 48 h (totaling 72 h of experiment), incubated under standard conditions until effluent collection and subsequent analysis.

### 2.5 Cellular viability

To assess the culture viability for both physiological and pathological set-ups, cells were manually counted in a Neubauer chamber. Culture medium was removed, and a 2 mg/mL collagenase-P (Roche, 11213857001) was injected into the fluidic ports until filled. The microdevice was incubated for 30 min at 37 ° C. After complete dissolution, the cells/collagen mixture was collected and stored in 300 μL DMEM/F12 15% FBS. For the adherent cells recovery, a trypsin/EDTA (Gibco, 25200072) solution was injected into channels, and the microdevice was incubated for 5 min. The supernatants were collected and stored together with the cells/collagen mixture. The solution was centrifuged, 300 *g* for 10 min at 4 ° C, the supernatant discarded, and resuspended in 200 μL DMEM/F12 15% FBS. This final solution was diluted 1:1 (v/v) in trypan blue, homogenized, and deposited into a chamber for quantification.

### 2.6 Culture organization and heterogeneity

Cell niche formation and distribution were assessed by pre-stained endothelial cells and osteoblasts with ${\text{CellTracker}}^{\text{TM}}$ Red CMTPX (1 μM ), while stromal and leukemic cells were stained with ${\text{CellTracker}}^{\text{TM}}$ Blue CMAC (10 μM ) and Green CMFDA (5 μM ) (Thermo Fisher), respectively, and co-cultured on the microdevice.

The 3D culture organization was evaluated using the Calcein-AM solution (Invitrogen, C3100MP) at 2 μ M. Staining was performed for 1 h at 37 ° C in serum- and phenol red-free RPMI, followed by gentle washing and resuspension in culture medium.

After 72 h of culture, samples were imaged under a fluorescence confocal microscope (Nikon–C2+), using a 10X magnification objective. The following excitation/emission filters were applied: 353/466 nm for Blue CMAC, 577/602 nm for Red CMTPX, 492/517 nm for Green CMFDA, and 494/517 nm for Calcein-AM. Image analysis and visualization were performed using NIS-Elements Viewer software (Nikon).

### 2.7 Phalloidin staining of F-actin

To confirm endothelial and osteoblast monolayer formation, cells were stained for F-actin after 72 h of culture. Culture medium was removed, and microdevices were washed twice with pre-warmed (37 °C) 1X PBS. Cells were fixed with 4% paraformaldehyde (Sigma, 158127) for 20 min at room temperature, followed by two washes with 1X PBS. Permeabilization was performed with 0.1% Triton X-100 (Sigma, X100) in PBS for 20 min. Samples were then blocked with 3% BSA (Sigma, A7906) in PBS for 1 h at 4 °C. After washing, cells were incubated with Alexa Fluor 488-phalloidin (Thermo Fisher, A12379) 1:400, for 1 h at room temperature. Following 1X PBS washes, nuclei were stained with DAPI (Thermo Fisher, 62247) 5 μg/mL for 20 min. Microdevices were maintained in PBS, and images were acquired using ${\text{EVOS}}^{\text{TM}}$ M3000-Invitrogen fluorescence imaging system (Invitrogen) with a 20X objectives.

### 2.8 Immunoassays

Cytokine, chemokine, and growth factor levels were quantified using the Bio-Plex ${\text{Pro}}^{\text{TM}}$ Human Cytokine 27-plex Assay (Bio-Rad). Concisely, the supernatants were collected in conical microtubes after 72 h of experiment (24 h in static and 48 h in perfusion), and centrifuged (1,174 g; 10 min; 4 ° C) to remove cell debris. Then, they were stored at −20 ° C until analysis. Samples were incubated with magnetic beads conjugated to specific antibodies for each ones analytes: FGF basic, Eotaxin (CCL11), G-CSF, GM-CSF, IFN- γ , TNF- α , IL-1 β , IL-1ra, IL-2, IL-4, IL-5, IL-6, IL-7, IL-8, IL-9, IL-10, IL-12 (p70), IL-13, IL-15, IL-17A, IP-10 (CXCL10), MCP-1 (CCL2), MIP-1 α (CCL3), MIP-1 β , PDGF-BB, CCL5, and VEGF. Detection was achieved via streptavidin-phycoerythrin. Data acquisition was performed on a Bio-Plex 200 system (Bio-Rad) and analyzed with Bio-Plex Manager v6.0 (Bio-Rad Laboratories, Hercules). At least 50 beads were acquired for each one. The final concentration (pg/mL) was obtained according to standard curves using a five-parameter logistic fit regression to convert the mean fluorescence intensities into picograms per milliliter.

Comparatively, each leukemic sample size was divided by the mean value of the healthy control group, and relative expressions were expressed as fold change (pathological/physiological). Fold change values greater than 1 indicate higher levels in the leukemic group, whereas values below 1 indicate lower levels relative to healthy controls. Statistical analyses were performed on absolute concentration values (pg/mL) .

### 2.9 Statistical analysis

Statistical analyses were performed using GraphPad Prism (version 8.0.2). Data are presented as mean ± standard deviation (SD). Independent groups were performed using an unpaired Student’s t-test. A *p*-value ≤ 0.05 was considered statistically significant.

## 3 Results and discussion

### 3.1 Native bone marrow microenvironment inspires microengineered biomimetic on-chip platform

**FIGURE 1:**
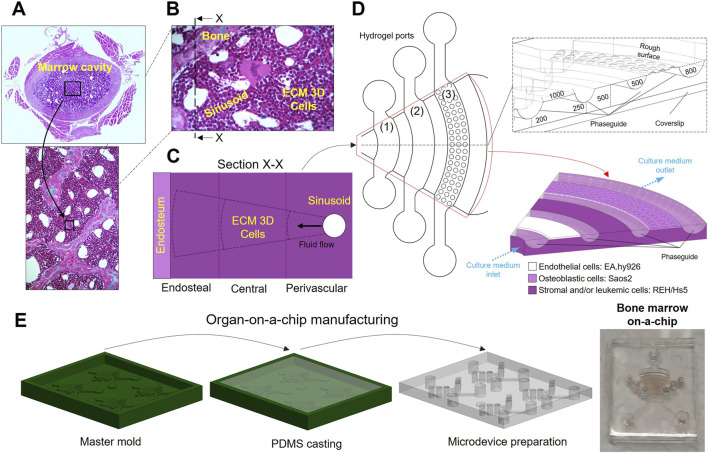
Conceptual biological framework and microengineered implementation of the bone marrow-on-a-chip platform. **(A)** Schematic representation of human bone marrow histology. **(B)** Structural organization of the medullary cavity subunits, highlighting trabecular bone, vascular structures, and stromal matrix. **(C)** An OoC composed of niches, sinusoids, and a 3D ECM. **(D)** Geometrical and experimental features of microengineered bone marrow-chip solution. Dimensions are presented in micrometers. **(E)** Microfabrication workflow of the bone marrow-on-a-chip: master mold fabrication by high-resolution 3D printer; PDMS casting via soft lithography; demolding, cutting, and port creation using biopsy punch; and bonding to glass coverslips through oxygen plasma. Histological image credits: (Boston University, 2002).

The bone marrow structure and function critical regulates in leukemic cell fate through niche-specific signaling, including cell-cell communication inside each niche and inter-niche cell communication mediated by soluble factors (Soto et al., 2021; Huang et al., 2024). To recapitulate the pathological site, our platform was inspired by the key features of the vascularized architecture of the medullary cavity (Figures 1A,B), which can be mainly represented by a sinusoidal vessel embedded in a collagen-rich ECM, lining the marrow and bone surface (Figure 1C).

Guided by marrow niches concept (Belyavsky, 2019), the biomimetic OoC comprises three specifics culture chambers: (1) a perivascular chamber, characterized by endothelial cells on upper wall of the microdevice exposed to an interstitial flow source; (2) a central chamber; simulating the core of the marrow stromal compartment, and (3) an endosteal chamber, with osteoblasts cultured on a rough-pattern surface, mimicking the bone interface (Figure 1D).

**FIGURE 2:**
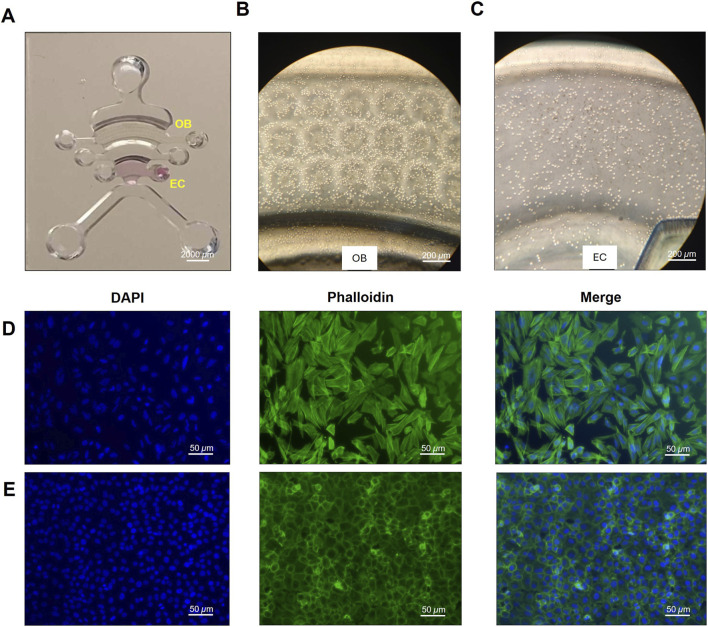
Cellular loading and organization in endosteal and perivascular compartments of the bone marrow-on-a-chip. **(A)** Representative image of cell loading into the osteoblastic (OB) and endothelial (EC) chambers. **(B)** Representative image of osteoblastic cells after loading into the endosteal compartment. **(C)** Representative image of endothelial cells after loading into the perivascular compartment. To assess monolayer formation on the device surface, cells were stained for F-actin and DAPI. Representative images are shown in **(D)** for osteoblasts and **(E)** for endothelial cells after overnight culture on the device surface. Images were acquired at 20X magnification.

Representative images illustrating cell loading and the initial spatial distribution of osteoblastic and endothelial cells immediately after seeding are shown below (Figures 2A–C). Monolayer formation of osteoblasts and endothelial cells after overnight culture is shown in Figures 2D,E, respectively.

Each microengineered chamber (1000 μm wide and 500 μm height) is interconnected by horizontal phaseguide structures, modeled via the Young–Laplace equation for pressure difference at the fluid-air interface for advancing fluid (Wang et al., 2016; Garbarino et al., 2017). These phaseguide-based features enable independent hydrogel loading while preventing leakage between compartments, allowing the establishment of compartmentalized, niche-specific cell cultures within an integrated microfluidic system under interstitial perfusion.

Building upon its architectural and functional bioinspired model, the platform was designed to study the dynamics of human leukemia within a biomimetic marrow *in vitro*. The microdevice contains three collagen hydrogel compartments: a perivascular niche seeded with endothelial and stromal cells, a central ECM with stromal cells, and an endosteal compartment with osteoblast and stromal cells (Figure 1D).

To model the pathological B-ALL condition, the B-cell precursor acute lymphoblastic leukemia (BCP-ALL) cell line REH and the stromal cells were embedded in the collagen hydrogel of all 3 compartments. Although recent marrow-on-a-chip models for ALL increasingly incorporate primary or patient-derived cells to better capture bone marrow heterogeneity, we deliberately employed well-established and physiologically relevant cell lines in our study. While a limitation, this choice enabled the development and validation of a robust, reproducible, and well-controlled OoC platform for initial investigations of cell-cell interactions and microenvironmental signaling in bone marrow. This strategy aligns with the Focus Group OoC Standardization Roadmap - 2024 (CEN/CENELEC, 2024), which recognizes immortalized cell lines as a valid cellular source for OoC development and validation.

From the biomimicry of microarchitecture and components of the ECM, and the spatial and temporal arrangement of cellular types and secreted biomolecules (Zhang et al., 2019; Ingavle et al., 2019; Pinho and Zhao, 2023), the pathological marrow was replicated in a bone marrow-on-chip, enabling the study of leukemic behavior in a spatial compartmentalized system. In summary, this bioengineered platform allowed us to interrogate the crosstalk between leukemic and endothelial, marrow stromal, and osteoblast cells. Moreover, it can provide insight into mechanisms that may contribute to tumor chemoresistance during B-ALL development and progression.

### 3.2 *In silico* model predicts physiologic interstitial fluid flow

**FIGURE 3:**
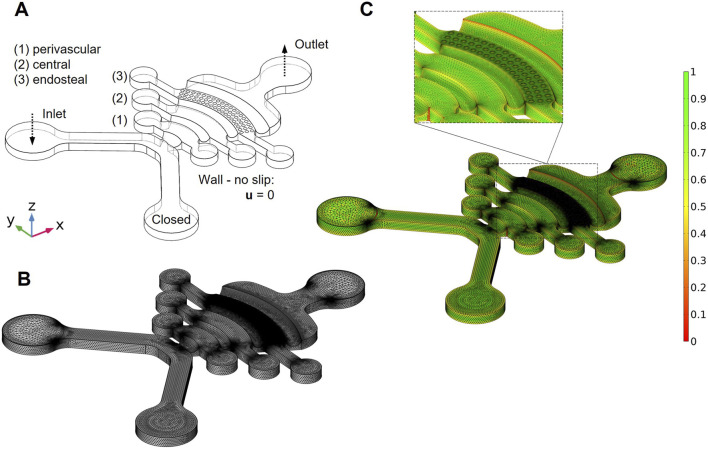
Computational microfluidic model. **(A)** Representation of the control volume and boundary conditions. Three regions mimic distinct physiological niches: (1) perivascular, (2) central, and (3) endosteal. Fluid is introduced through the inlet at 0.05 μL/min . A non-slip condition (**u** = 0) was adopted in the channel walls. **(B)** Visualization of the 3D mesh used in simulations, highlighting the high resolution in the curvature regions and transitions between chambers. **(C)** Distribution of the mesh skewness parameter along the microdevice, ranging from 1 (perfectly regular, in green) to 0 (degenerated, in red). The image shows the uniformity of the mesh and the absence of highly distorted elements.

The primary goal of this work was to integrate experimental OoC cellular culture and computational microfluidics. For this purpose, an *in silico* model based on fluid dynamic equations was implemented to evaluate the perfusion within the OoC (Figure 3A). To assess the robustness of the computational model, a grid convergence analysis was performed using three levels of free tetrahedral meshes: Extra Fine (4,158,058 elements), Finer (1,531,133 elements), and Fine (607,317 elements).

The average interstitial velocities were measured at the centered point for each culture chamber (perivascular, central, and endosteal). For each region, the indices ${\text{GCI}}^{21}$ and ${\text{GCI}}^{32}$ decreased substantially when the mesh was refined: in the perivascular chamber, from 1.38% to 0.12%; in the central chamber, from 1.34% to 0.49%; and in the endosteal chamber, from 2.83% to 0.38%. These results show that from *Finer* to *Extra Fine* mesh ( ${\text{GCI}}^{32}$ ) numerical velocity will not change significantly, indicating a mesh-independent simulated (Roache, 1994) for a refinement at a *Finer* mesh, which conditioned its use. This tetrahedral mesh is composed of elements with sizes ranging from 0.0111 to 0.102 mm. An extra refinement in the corners and boundary layers (Figure 3B) resulted in an average shape skewness of 0.6821 (Figure 3C). This skewness value is within the range considered acceptable for finite elements solutions and indicates the metric quality of the elements in the mesh (Ansys Inc, 2025).

Working with both finite element methods to evaluate the mesh quality, skewness, element metric that is related to precision and simulation stability, and GCI, an index that determines the grid accuracy, a mesh was created that mitigates the induced uncertainty in the velocity profile and distribution within the geometry. This established that the computational fluid dynamics model was able to replicate *in silico* the physiological interstitial fluid flow as an *in situ* marrow microenvironment, allowing it to serve as a guide for the definition of the ideal boundary conditions and experimental configuration for proposed use.

**FIGURE 4:**
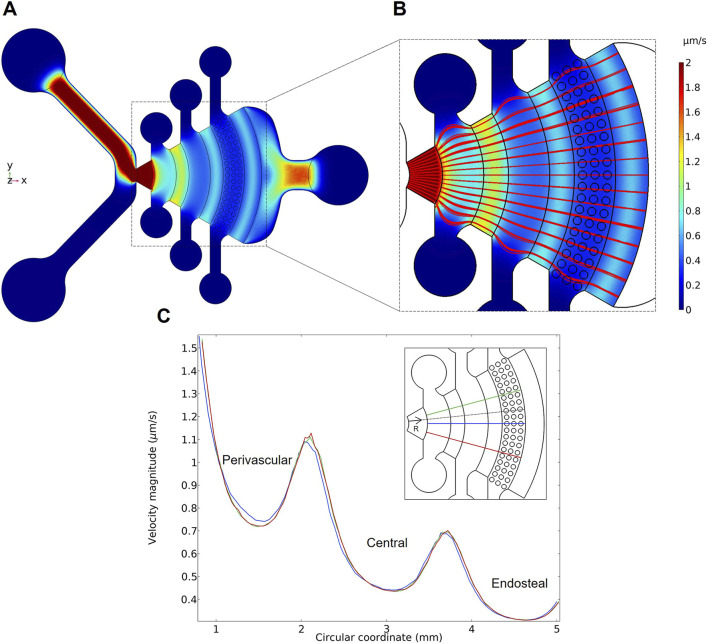
Velocity field and comparative flow analysis in the three functional regions of the bone marrow-chip. **(A)** Spatial distribution of velocity magnitude in the *xy*-plane of the microfluidic microdevice. **(B)** Enlargement of the semicircular area, highlighting the streamlines and the detailed velocity profile. Velocity field: distribution of velocities in the *xy*-plane and streamlines. The colors represent the velocity intensity (0–2 μm/s ), showing greater velocity in the inlet channel and progressive decrease as the fluid disperses in the perivascular, central, and endosteal regions. **(C)** Velocity magnitude profiles along three radial trajectories (highlighted in the inset) that traverse the perivascular, central, and endosteal regions. The three profiles show similar behavior, evidencing velocity peaks and homogeneity along the valleys. R stands for circular coordinate reference.

Using *in silico* simulations, flow generated by a syringe pump was tested until interstitial fluid velocity within the chambers culture reached values on the order of $10^{-1}\mu m/s$ , as expected for bone marrow physiology (Dafni et al., 2002). An unilateral inlet configuration was proposed to minimize the risk of bubble formation and hydrogel displacement, two critical factors for maintaining culture integrity (Sung and Shuler, 2009). The fluidic channel was filled with culture medium via a single inlet, and the opposite fluidic port was closed, directing the hydraulic pathway to the culture chambers. This boundary flow set-up produced a symmetric velocity in the *xy*-plane (Figure 4A), measured at a representative height of 125 μm above the chamber base. Streamlines analysis confirmed a stable laminar flow pattern (Figure 4B), ensuring that the cells situated in the same culture chambers experience uniform hydrodynamic stimuli.

Due to the circumferential-based geometry of chambers in bone marrow-on-chip, the average fluid velocity decreased progressively from perivascular to endosteal chambers, with values of: 0.77 μm/s , 0.44 μm/s , and 0.30 μm/s , respectively (Figure 4C). Furthermore, localized velocity peaks were observed adjacent to the phaseguide, reaching 50%–60% above the mean chamber velocity. Despite these localized peaks, the velocities in the culture remained within the physiological range expected for interstitial flow in bone or bone marrow tissues (Hillsley and Frangos, 1994).

The developed design introduces an alternative conceptualization of a capillary burst valve (phaseguide-based gates). Conventionally, this valve is constructed using ports (Shin et al., 2012; Polacheck et al., 2014), vertical structures that confine the hydrogel filling in specific regions. For these traditional designs, the flow remains nearly invariant across the cross section, except for the non-slip condition at the walls. The proposed design induces variations in velocity along the *xz*-plane due to the narrowing of the effective cross sectional area near the phaseguide. This effect is evident in regions adjacent to the gates (Figure 4).

**FIGURE 5:**
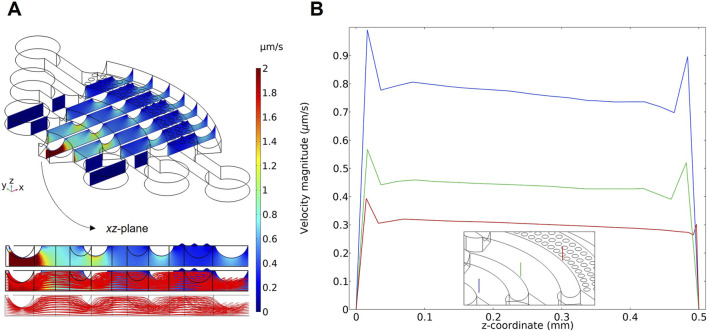
Evaluation of the velocity field along the *xz*-plane and analysis of the vertical flow profile in the three functional regions of the bone marrow-chip. **(A)** Distribution of velocity magnitude along consecutive sections in the *xz*-plane, enabling visualization of flow variation along the vertical (*z*) axis. Cross-sectional analyses indicate that velocity remains nearly uniform along the *z*–axis, except in the vicinity of the phaseguided structures. Streamlines representations confirm laminar flow throughout the full height of the device. **(B)** Vertical profiles of velocity magnitude along the (*z*-direction) obtained at three representative locations: perivascular (blue line), central (green line), and endosteal (red line) regions, as indicated in the inset. All regions display a pronounced reduction in velocity near the channel walls, consistent with the no-slip boundary condition, followed by an approximately constant plateau across the channel height. The perivascular region exhibits higher peak velocities, whereas the endosteal region shows comparatively lower values, reflecting geometric constraint and differential flow distribution among the distinct microenvironmental niches.

In accordance with the velocity field results (Figure 4C), although the flow remains symmetric across the circumferential axis of each chamber, a variation was observed mainly in sites adjacent to the gates (Figure 5A). Streamline analysis confirmed the absence of vortex formation or stagnant zones, which could otherwise lead to inhomogeneous stimulation of the cells.

Regarding each culture chamber, velocity along the *z*-axis exhibited shows a slight internal heterogeneity, with values ranging from 0.805 to 0.698 μm/s in the perivascular region; 0.459 to 0.391 μm/s in the central region; and 0.321 to 0.265 μm/s in the endosteal region (Figure 5B). These flow profiles are characteristics of porous media under low Darcy number conditions $\left(\kappa /h^{2}\approx 10^{-6}\right)$ .

Within the bone marrow microenvironment, biophysical cues, including ECM composition, mechanical stiffness, and the interstitial fluid shear stress, serve as critical modulators of cell behavior. In particular, shear stress levels in the interstitium are lower than those observed in the bloodstream (Rutkowski and Swartz, 2007). Under physiological conditions, interstitial fluid exposes stromal cells to shear stresses ranging from 0.01 to 0.1 Pa, values several orders of magnitude below those experienced by cells in arterial circulation (Kim et al., 2014). Nevertheless, even at these relatively low levels, interstitial shear stress is sufficient to modulate cell phenotype and function.

Leukemic cells have demonstrated increased chemotherapeutic resistance when exposed to microenvironments that replicate both physiological matrix stiffness and fluidic shear stress (Bruce et al., 2015). In our model, the velocity fields predicted by computational simulation replicate these *in vivo*-like mechanical conditions, which are responsible for regulating the cellular phenotype and functions, through mechanotransduction and mechanosensation mechanisms (Christoffersson, 2018). Therefore, cells in each compartment present a homogeneous behavior, enabling both individual and comparative studies of cellular functions within the distinct niches of the bone marrow-on-chip platform.

Although experimental assessment remains essential, the *in silico* framework provides predictive insights into fluid dynamics within an on-chip platform. As such, computational modeling serves as a strategic tool for optimization and improvement, performing a key role in the design of this new platform to study bone marrow pathophysiology *in vitro*.

### 3.3 Bone marrow-on-chip recreates niches and supports maintaining pathophysiological cells

To determine the extent to which the *in vitro* chip recapitulates the cellular heterogeneity and spatial organization of the human bone marrow microenvironment *in vivo*, we analyzed niche-specific cell distribution and colocalization using ${\text{CellTracker}}^{\text{TM}}$ fluorescent labeling (Thomas et al., 2023). For both control and leukemic conditions, distinct niche-specific cellular combinations were established, with each cell population identified by a defined fluorescent label.

In the perivascular compartment, the control configuration included endothelial cells (red) and stromal cells (blue), whereas the leukemic configuration comprised endothelial and stromal cells together with leukemic cells (green) under leukemic conditions. In the endosteal compartment, the control setting consisted of osteoblasts (red) and stromal cells, while the leukemic configuration incorporated osteoblasts, stromal cells, and leukemic cells.

**FIGURE 6:**
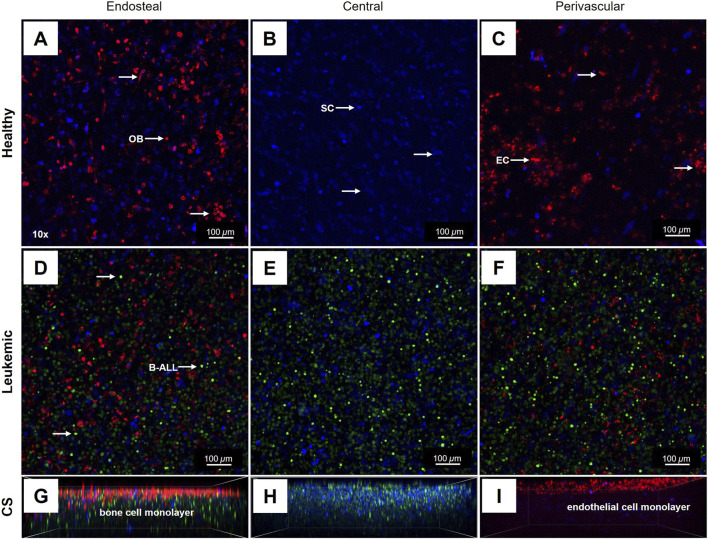
Representative images of cell co-culture in the bone marrow-on-chip device. Healthy marrow: **(A)** Red-labeled osteoblasts (OB) in co-culture with blue-labeled stromal cells (SC) in the endosteal niche. **(B)** Stromal cells cultured in the central niche. **(C)** Red-labeled endothelial cells (EC) in co-culture with stromal cells in the perivascular niche. Leukemic marrow: **(D)** Green-labeled leukemic cells interacting in tri-culture with osteoblasts and stromal cells in the endosteal niche. **(E)** Co-culture of leukemic and stromal cells in the central niche. **(F)** Interaction between leukemic, stromal, and endothelial cells in the perivascular niche. **(G)** Co-location of leukemic, stromal cells, and osteoblast monolayer. **(H)** 3D arrangement of B-ALL and stromal cells. **(I)** Endothelial monolayer and stromal cells 3D distribution. Images were acquired using a confocal microscope after 72 h of culture (24 h in static and 48 h in perfusion) at 10X. Biological replicates with n = 4. OB = osteoblasts, SC = stromal cells, EC = endothelial cells, B-ALL = leukemic cells, CS = cross section.

Collectively, these configurations demonstrate that the proposed chip was able to create a niche-based cellular compartmentalization, bounded by a membraneless intact collagen gel. These features address current gaps in biomimicry, potentially leading to a biologically relevant *in vitro* platform that can recapitulate key structural and organizational features of the native bone marrow microenvironment. Across all compartments, cells exhibited spatially homogeneous and tissue-like distributions (Figures 6A–F).

Confocal microscopy of the dynamic 3D cultures, under both healthy and leukemic conditions, revealed potential cell-cell interactions, highlighting the close spatial association between leukemic cells and the structural components of each niche (Figures 6D–F). F-actin staining further showed well-defined adhesion and cellular organization of endothelial and osteoblastic cells along the microengineered regions. (Supplementary Figure S1A,B).

Osteoblasts (OB; red fluorescence) and endothelial cells (EC; red fluorescence) (Figures 6A,C,D,F) are attached to the device surface. Stromal cells (SC; blue fluorescence) exhibited an elongated and pseudo-spherical morphology, although rounded conformations were also noticed (Figure 6B). Notably, both rounded and elongated phenotypes were frequently observed within the same field of view, indicating intracompartimental heterogeneity.

Cell morphology in 2D and 3D environments is strongly influenced by ECM composition, stiffness, and mechanical cues mediated by mechanosensitive pathways (Ross et al., 2012; Ivanovska et al., 2015). In general, softer matrices promote rounded morphologies, whereas stiffer substrates promote cell spreading and elongation (Nashchekina et al., 2017; Galarza Torre et al., 2018; Zhang et al., 2019; Geiger et al., 2019; Hou et al., 2020; Alamán-Díez et al., 2023; Park et al., 2023). In collagen-based matrices with stiffness values around 300 Pa, bone marrow-derived mesenchymal stromal cells have been reported to exhibit partially elongated morphologies (Polacheck et al., 2013; Bruce et al., 2015). Similarly, previous studies in 3D systems under continuous flow conditions have shown the coexistence of rounded and elongated phenotypes within the same microenvironment (Bruce et al., 2015). Consistent with these reports, our results show the coexistence of rounded and elongated cells within the same 3D microfluidic device (Supplementary Figure S1, S2).

**FIGURE 7:**
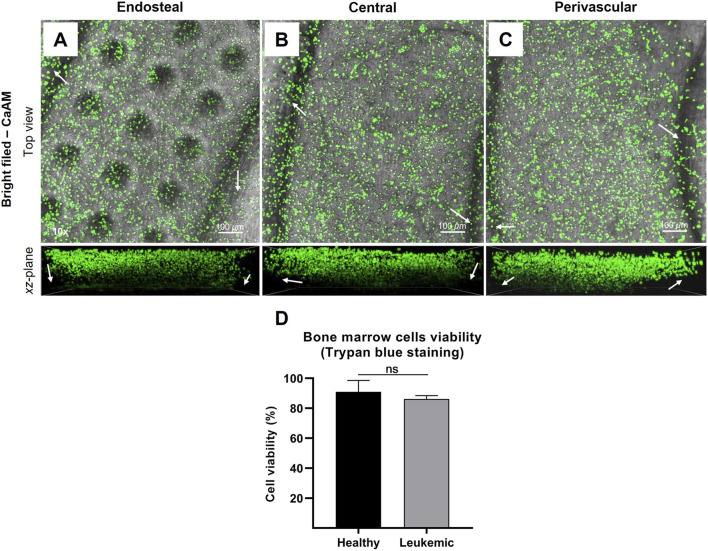
Representative images of spatial distribution, physical and biological integration in the bone marrow-on-chip, and cell viability. Cells were labeled with green calcein-AM solution, and the images were acquired using a confocal microscope after 72 h of culture (24 h in static and 48 h in perfusion) at ×10 magnification: **(A)** Endosteal niche. **(B)** Central niche. **(C)** Perivascular niche. Integration of adjacent niches, forming a continuous cellular microenvironment. White arrows point to the phaseguide structures and the interconnected regions of culture chambers. **(D)** Cells were recovered from the microdevice, and the viability of the leukemic and healthy groups was determined by trypan blue staining at 72 h. Biological replicates with n = 4. Statistical analyses were performed using GraphPad Prism (v. 8.0.2). Means ± SD independent groups were performed using an unpaired Student’s t-test (p ≤ 0.05).

Cross-sectional images (Figures 6G,I) revealed that both bone and endothelial cells organized in an upper layer along the chamber surfaces, biomimicking *in vitro* the microstructure of the endosteum surface and vessel wall. Vertical cell distribution was also confirmed along the chamber’s height (Figure 6H). Three-dimensional organization of the diverse cell populations used in the system (bone, endothelial, stromal and leukemic) was evaluated using Calcein-AM staining. Fluorescence imaging confirmed cellular distribution within the collagen matrix, both within individual compartments and across adjacent niche regions (Figure 7).

Specifically in the perivascular-central (Figures 7B,C) and central-endosteal (Figures 7A,B) regions, the presence of cells was observed in the respective culture chambers and along the phaseguide structures, between the regions. Cellular presence along the phaseguide structures further supports the formation of an integrated, compartmentalized microenvironment that enables multicellular interaction and signaling across tissue-like barriers.

The successful implementation of an integrated microenvironment using a phaseguide-based confinement approach provides substantial advantages over traditional pillar-based systems. The geometry used to confine hydrogel plays a key role in cellular behavior, affecting their phenotype by interaction with the microdevice surface (Lampin et al., 1997). In pillar-based systems, structural posts may create a tough path for migrating through the space between them and the gel. This limitation is particularly relevant in monolayer cultures, on the gel-media interface (Loessberg-Zahl et al., 2020). The pillar approach can negatively affect the integrity of the monolayer, impairing the establishment of physiological levels of relevant biological cues (van Duinen et al., 2015). In this context, the proposed design overcomes these limitations by enabling continuous culture zones and structure in microchannel microvessels-like barriers (Beaurivage et al., 2019; Poussin, 2020; Riddle et al., 2022).

To verify the ability of the platform to support the pathophysiological cell co-culture, in a biocompatible marrow environment, its efficiency in maintaining a viable cellular culture for 72 h was quantified. Cell counts included the entire multicellular population within the microdevice. The results show that cells maintained under physiological conditions exhibited an average overall viability of 92%, whereas those under pathological conditions showed 86% viability (Figure 7D).

In this experiment, both biological set-ups maintained viability above the 80% threshold (Torisawa et al., 2014), a value commonly adopted as a reference for metabolically active and functional cell cultures (Segeritz and Vallier, 2017). Importantly, comparable baseline viability was observed between the control and leukemic configurations, suggesting that the platform provides a stable cellular environment suitable for comparative analyses, including potential pharmacological studies.

In summary, these results demonstrate that the designed advanced platform enables compartmentalized 3D culture system under continuous interstitial flow, maintaining spatially organized co-cultures and supporting interactions between niche cells and REH leukemic cells (Figures 6, 7). This organization is achieved through phaseguide-mediated compartimentalization, which allows independent hydrogel loading and stable spatial separation of cell populations and controlled culture conditions.

### 3.4 Biomimetic chip recapitulates cell-signaling modulation induced by leukemic cells

The proposed bone marrow-on-a-chip recreates *in vitro* the integrated nature of bone marrow niches, enabling reproduction of cell-cell and cell-matrix interactions in co-culture. Beyond structural mimicry, this system allows investigation of B-ALL-driven modulation of microenvironment via cytokines, chemokines, and growth factors, key mediators involved in stromal and hematopoietic support and leukemic progression (Tabe and Konopleva, 2014; Houshmand et al., 2019).

**FIGURE 8:**
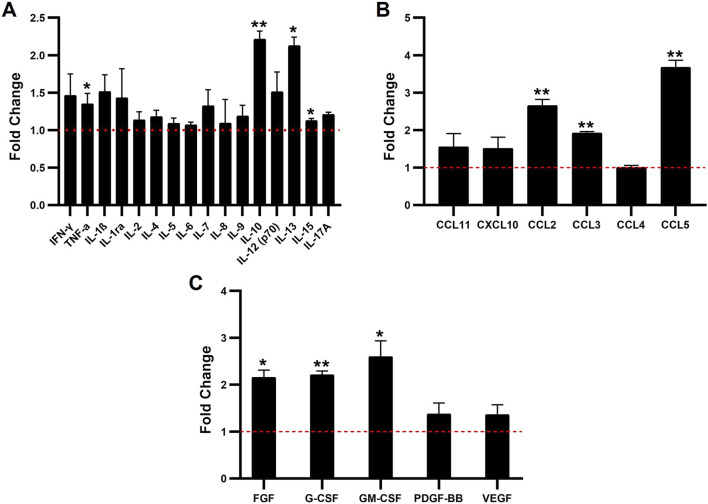
B-ALL cell with niche cells co-culture mimics cell-signaling modulation. **(A)** Fold change in cytokine levels. **(B)** Fold change in chemokine levels. **(C)** Fold change in growth factor levels. Fold change in the production of soluble factors obtained from the 72 h (24 h in static and 48 h in perfusion) supernatants of dynamic bone marrow-on-chip co-cultures subjected to the Luminex immunoassay. Each bar depicts the relative expression (fold change) of an individual soluble molecule. For each analyte, the mean concentration (pg/mL) in leukemic samples was normalized to the corresponding mean value in healthy controls (pg/ ${mL}_{\mathrm{leukemic}}$ /pg/ ${mL}_{\mathrm{healthy}}$ ). Values greater than 1 indicate increased expression, whereas values below 1 indicate reduced expression relative to the healthy group. The dotted line represents the baseline level of healthy controls. Biological replicates with n = 4. Graphs were generated in GraphPad Prism (v. 8.0.2), and the results are presented as means ± SD. IFN = interferon, IL = interleukin, FGF = fibroblast growth factor, G-CSF = granulocyte colony-stimulating factor, GM-CSF = granulocyte-macrophage colony-stimulating factor, PDGF = platelet-derived growth factor, VEGF = vascular endothelial growth factor.

To investigate whether the model reflects the immunomodulatory landscape of leukemic microenvironment, the soluble mediators implicated in B-ALL *in vivo* progression, resistance, and relapse were quantified (Hughes et al., 2022; Štajer et al., 2024). The cell culture supernatants were collected and subjected to the Luminex-Bio Rad 27 Plex enzyme immunoassay (Figure 8). Fold-change analyses were performed for cytokines, chemokines, and growth factors across leukemic and healthy (control) marrow-mimetic conditions.

Cytokine profiling revealed changes in the inflammatory mediator landscape in the presence of REH leukemic cells. Increased levels of cytokines commonly associated with Th1-related signaling, including TNF- α and IL-15, were observed, together with cytokines often linked to Th2-associated pathways, such as IL-10 and IL-13 (Yoshikai and Nishimura, 2000; Romagnani, 2000; Cavalcanti et al., 2012; Lee et al., 2019) (Figure 8A). These findings indicate the presence of cytokine mediators associated with both inflammatory signaling patterns within the system. Notably, cytokines typically linked to Th2-related pathways showed a tendency toward higher levels, suggestive of an immunoregulatory profile.

In anti-tumor responses, including B-ALL, this suggestive cytokine profile in the chip mirrors the permissiveness of leukemia development found *in vivo*, once the overexpression of IL-10 and IL-13 is associated with suppression of the activation of key immunocompetent cells, such as macrophages, neutrophils, and lymphocytes, in the tumor milieu. In concordance with the obtained results, data have shown elevated levels of IL-10, an immunoregulatory cytokine, in ALL pediatric patients and their strong association with disease severity and relapse risk (Ye et al., 2026). In addition, IL-13 has been implicated in tumor progression (Terabe et al., 2004) and in the protection of leukemic blasts from apoptosis (Chaouchi et al., 1996).

Supporting the translational relevance of these findings, the bone marrow microenvironment of pediatric B-ALL patients exhibits an immunosuppressed phenotype characterized by elevated IL-10 levels. Notably, therapeutic clearance of leukemic cells is associated with reversal of this immunosuppressive profile and a significant increase in IFN- γ expression (Magalhães-Gama et al., 2021).

Importantly, elevated TNF- α expression indicates the activation of an inflammatory signaling axis that sustains NF- κ B mediated anti-apoptotic and pro-survival pathways, thereby promoting tumor persistence, despite its original characterization as a tumoricidal cytokine (Karin, 2006). In leukemia, TNF- α drives disease progression by sustaining proliferation, angiogenesis, microenvironmental remodeling, immune evasion, and resistance to therapy (Zhou et al., 2017), and is increased at diagnosis in ALL patients, correlating with adverse clinical parameters (Verma et al., 2022; Ahmed and Al Hatem, 2024).

Concomitantly, IL-15 (a pro-inflammatory cytokine) upregulation indicates the establishment of a survival-supportive niche. Through several pathway activation (JAK/STAT, ERK, PI3K/AKT), this cytokine promotes proliferative and anti-apoptotic signaling in ALL. Its overexpression correlates with disease severity and central nervous system involvement and may influence minimal residual disease and treatment response (Sindaco et al., 2023). Ultimately contributing to reduced overall survival in patients with ALL (Gallardo Rodríguez et al., 2023).

Chemokines analysis confirmed a leukemic microenvironment enriched in pro-recruitment signals, with significantly high levels of Eotaxin MCP-1 (CCL2), MIP-1 α (CCL3), and RANTES (CCL5) (Figure 8B) compared to the healthy (control) environment. Leukemic niche-derived chemokines mediate immune cell recruitment to the bone marrow; however, concomitant elevation of immunoregulatory cytokines such as IL-10 and IL-13 reprograms these infiltrating cells into anti-inflammatory, pro-tumor phenotypes (e.g., M2 macrophages, N2 neutrophils, and Tregs), suppressing antitumor responses and promoting immune evasion and leukemic persistence.

In this context, our results show significantly increased expression of the chemokines CCL3 and CCL5 in the presence of leukemic cells within the biomimetic bone marrow model. These findings are consistent with previous studies reporting elevated levels of these chemokines in pediatric B-ALL patients and their association with poor prognosis. Consistent with our observations, previous studies have reported increased levels of the chemokines CCL3 and CCL5 in pediatric B-ALL patients, where their elevation has been associated with poor prognosis (Zheng et al., 2025). In addition the increase in CCL2 in ALL patients has been related to stromal support for leukemic cells (De Vasconcellos et al., 2011). Moreover, the presence of chemokines, such as CCL2 and CCL5, in the tumor milieu is associated with the activation of chemoresistance pathways in leukemic cells (Aldinucci et al., 2020; Modak et al., 2024). These findings are further corroborated by co-culture studies, reporting an increased chemokine secretion when leukemic cells interact with mesenchymal stromal cells (Smeets et al., 2020).

Secreted molecules that exert auxiliary pro-tumor functions, growth factors production was upregulated in the presence of B-ALL REH cells when compared to the healthy (control) group (Figure 8C). Elevated FGF2 levels in the leukemic bone marrow niche promote stromal cell proliferation and activate pro-survival signaling pathways. This contributes to microenvironmental remodeling that reinforces leukemia cell maintenance and therapeutic resistance. High levels of urinary bFGF were observed in children with newly diagnosed ALL compared with non-leukemic controls, further supporting the notion that FGF signaling is elevated in the leukemic context (Javidi-Sharifi et al., 2019). Similarly, increased levels of G-CSF and GM-CSF were found in the presence of B-ALL cells, indicating a coordinated remodeling of the bone marrow niche. More specifically, G-CSF and GM-CSF support leukemic progression by expanding myeloid-lineage elements and shaping a permissive immune microenvironment; notably, bone marrow mononuclear cells from ALL patients produce elevated amounts of these mediators, demonstrating that leukemic infiltration alters local cytokine output toward a pro-tumoral profile (Vilchis-Ordoñez et al., 2015; Hong, 2016).

A potential limitation of this analysis is the higher total cell number in the leukemia condition, which may influence protein levels in the supernatant. Thus, part of the increased cytokine levels may reflect cell number rather than per-cell secretion. However, previous study demonstrated that B-ALL cells cultured alone typically exhibit limited cytokine production, and that cytokine release in leukemic niches is largely driven by stromal interactions (Ma et al., 2020). Therefore, the observed changes here likely reflect, at least in part, microenvironmental interactions.

While structural remodeling and therapeutic responses were beyond the scope of the present study, the leukemia-induced alterations in soluble factors identified represent early and mechanistically relevant drivers of niche dysfunction. Such molecular perturbations are recognized as upstream events that precede architectural changes and influence disease progression within the marrow microenvironment (Hughes et al., 2022).

In summary, the bone marrow-on-a-chip was able to recreate some cell-signaling features associated with the B-ALL microenvironment, including a trend toward immunoregulatory cytokine fold-change expression patterns, increased chemokine indices linked to microenvironment remodeling that may favor leukemia establishment, and elevated growth-factor fold-changes suggestive of support for leukemic survival. These microenvironmental signatures show similarities to patterns described in poor-prognosis B-ALL, suggesting that the platform may capture aspects of clinically relevant tumor niche remodeling. By approximating features suggestive of immune modulation, stromal adaptation, and pro-survival signaling, this system provides a physiological tool that may contribute to drug testing and to the study of microenvironment-mediated resistance pathways in ALL.

## 4 Conclusion

In this work, we developed an experimental and computational bone marrow-on-a-chip platform that integrates microfluidic design, multicellular 3D culture in ECM-based hydrogels, and computational fluid dynamics. The system enables the spatial organization of structural and leukemic cells within compartmentalized regions under continuous interstitial flow, providing a controlled environment to investigate cell-microenvironment interactions under defined biophysical conditions.

A key strength of the platform lies in its phaseguide-mediated compartmentalization, which allows independent hydrogel loading and stable spatial separation of cell populations without the need for a solid membrane. This membraneless configuration supports continuous yet compartmentalized culture regions, preserving multicellular organization while enabling communication across adjacent niches. This approach offers improved control over *in vitro* niche formation and facilitates the investigation of leukemia-microenvironment interactions within a spatially defined architecture inspired by bone marrow organization.

The integration of computational modeling further strengthens the platform by enabling the prediction of flow distribution and shear conditions under perfusion, within ranges compatible with interstitial flow. This computational–experimental framework was instrumental in defining inlet configuration, chamber geometry, and phaseguide dimensions, supporting stable perfusion and controlled flow distribution across compartments.

Biologically, the system supported the maintenance of multiple cell types under both control and leukemic conditions and enabled the analysis of leukemic cell-microenvironment interactions in a spatially organized setting. The presence of B-ALL REH cells was associated with changes in soluble mediator profiles, including cytokines, chemokines, and growth factors related to inflammatory signaling, stromal activation, and leukemic cell support, particularly IL-10, IL-13, TNF-α, IL-15, CCL2, CCL3, CCL5, FGF, G-CSF and GM-CSF upregulation. These findings are consistent with signaling patterns described in human leukemic bone marrow microenvironments and indicate that the platform captures relevant aspects of leukemic niche-associated remodeling.

By combining compartmentalized architecture, membraneless design, and CFD-informed perfusion control, the system contributes to the development of more advanced microphysiological models of hematological malignancies. Overall, the proposed leukaemia-on-a-chip provides a robust and versatile platform for studying bone marrow microenvironment interactions and may support future investigations into microenvironment-mediated mechanisms and the evaluation of novel therapeutic agents.

## Data Availability

The original contributions presented in the study are included in the article/Supplementary Material, further inquiries can be directed to the corresponding author.
