# Supplementary material for: A microfluidic bone marrow model combining CFD and organ-on-a-chip technologies to study leukemia niche dynamics
Source: Front Bioeng Biotechnol. 2026 May 28;14:1766296. doi: 10.3389/fbioe.2026.1766296 (PMC13253809; doi:10.3389/fbioe.2026.1766296)
Supplement: Supplementary file 1 [file Image1.pdf]

Supplementary Material

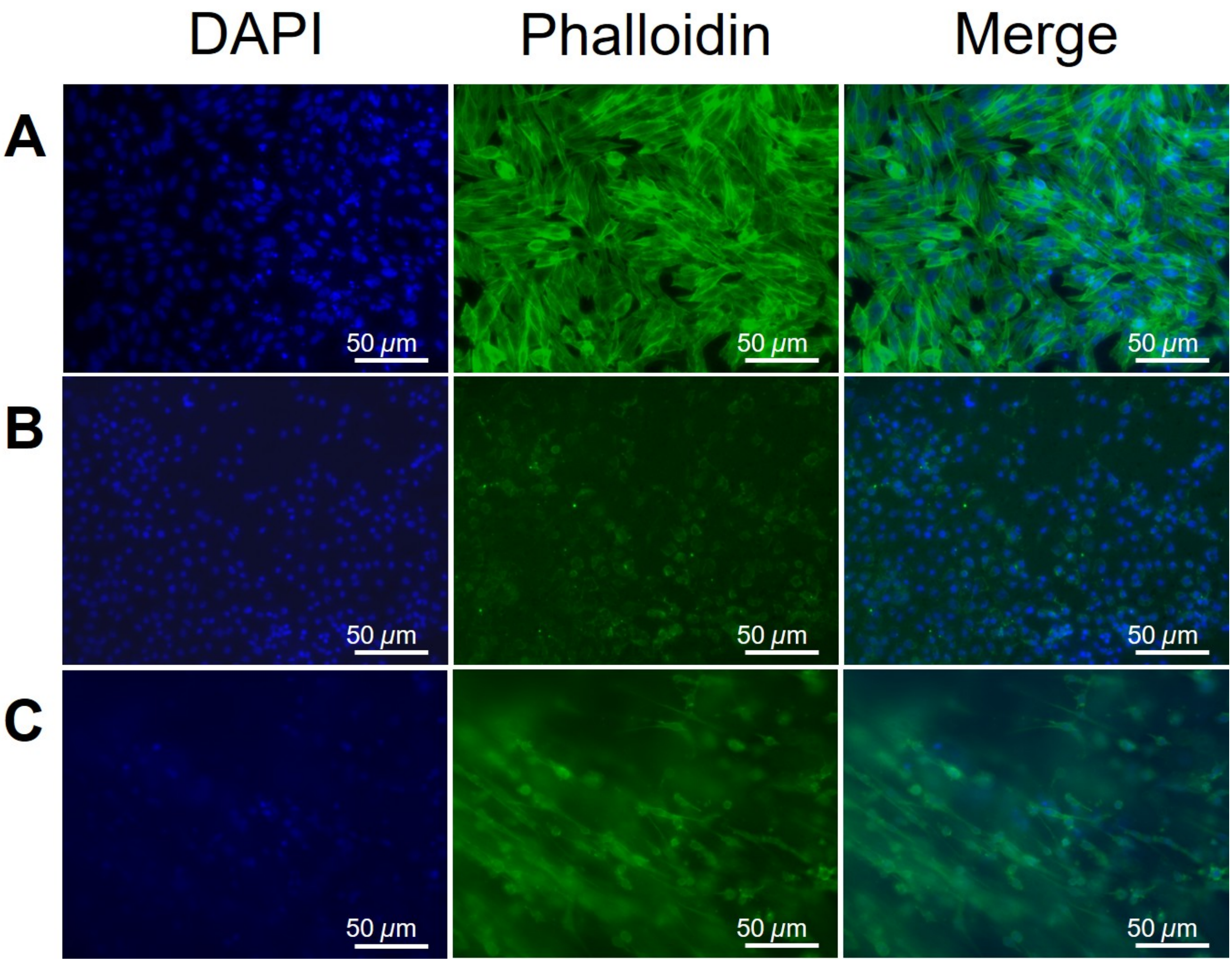

Figure S1. Cells were stained with DAPI and phalloidin (F-actin) to assess monolayer formation on the microdevice surface. Representative images are shown in (A) for osteoblasts and in (B) for endothelial cells after 72 hours (24 hours in static and 48 hours in perfusion) on the microdevice surface. In (C), stromal cells exhibiting elongated and pseudo-spherical morphologies were visualized within a 3D hydrogel under interstitial flow conditions. All images were acquired at 20X magnification.

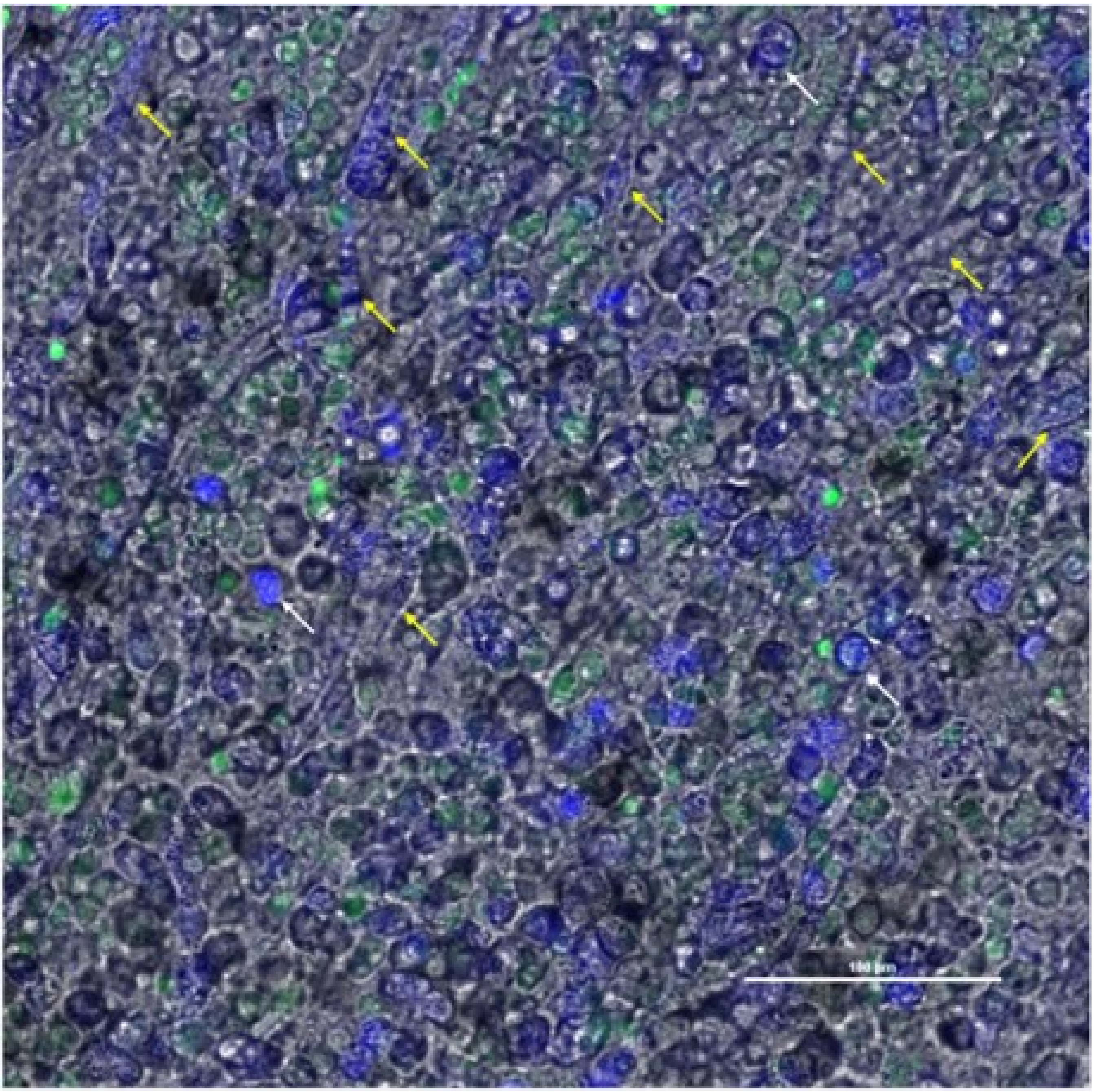

Figure S2. Representative confocal brightfield images showing heterogeneous stromal cell morphologies in the bone marrow-on-chip device. Stromal cells (blue-labeled) and B-ALL REH cells (green-labeled) were co-cultured in the central niche and imaged after 72 hours (24 hours in static and 48 hours in perfusion) of culture at 30X magnification. Yellow arrows indicate elongated stromal cells, while white arrows indicate rounded stromal cells.
